# Alterations of the Temporomandibular Joint on Magnetic Resonance Imaging according to Growth and Development in Schoolchildren

**DOI:** 10.1155/2012/695136

**Published:** 2012-12-19

**Authors:** Tatsurou Tanaka, Tetsuro Konoo, Manabu Habu, Masafumi Oda, Shinji Kito, Masaaki Kodama, Shinya Kokuryo, Nao Wakasugi-Sato, Shinobu Matsumoto-Takeda, Ikuko Nishida, Kazumasa Morikawa, Katsura Saeki, Kenshi Maki, Kazuhiro Tominaga, Shin-ichi Masumi, Masamichi Terashita, Yasuhiro Morimoto

**Affiliations:** ^1^Department of Oral Diagnostic Science, Kyushu Dental College, 2-6-1 Manazuru, Kokurakita-ku, Kitakyushu 803-8580, Japan; ^2^Department of Clinical Communication and Practice, Kyushu Dental College, 2-6-1 Manazuru, Kokurakita-ku, Kitakyushu 803-8580, Japan; ^3^Department of Oral and Maxillofacial Surgery, Kyushu Dental College, 2-6-1 Manazuru, Kokurakita-ku, Kitakyushu 803-8580, Japan; ^4^Department of Growth and Development of Function, Kyushu Dental College, 2-6-1 Manazuru, Kokurakita-ku, Kitakyushu 803-8580, Japan; ^5^Department of Oral Functional Reconstruction, Kyushu Dental College, 2-6-1 Manazuru, Kokurakita-ku, Kitakyushu 803-8580, Japan; ^6^Center for Oral Biological Research, Kyushu Dental College, 2-6-1 Manazuru, Kokurakita-ku, Kitakyushu 803-8580, Japan

## Abstract

The paper explains the alterations of the temporomandibular joint (TMJ) visualized by magnetic resonance imaging (MRI) according to the growth and development of schoolchildren. Appearance and disappearance of a “double contour-like structure” (DCLS) of the mandibular condyle on MRI according to the growth and development of schoolchildren were demonstrated. In addition, possible constituents of DCLS and the significance of detection of DCLS on MRI were also speculated. The relationship between red marrow and yellow marrow in the articular eminence of temporal bone, the disappearance of DCLS, and alterations of the mandibular condyle have been elucidated.

## 1. Introduction

Magnetic resonance imaging (MRI) has been extensively used to noninvasively evaluate various tissues as it can provide high resolution for the differentiation between different soft tissues. For various kinds of joints, including the temporomandibular joint (TMJ), the clinical application of MRI has led to better understanding of anatomy, growth, and disease [1–15]. Alterations in MRI signals corresponding with the growth and development of joints in infants and children have been well documented [2–15]. In particular, changes in normal cartilaginous epiphyses, physes, and metaphyseal marrow during growth have been evaluated extensively using MRI [2–7, 9–11], as it is difficult to obtain the precise imaging of these tissues by computed tomography (CT) [16, 17].

The growth and development of mandibular condyles and the articular eminence have been studied with regards to TMJ in the oral and maxillofacial region in schoolchildren [1, 9–11]. In consecutive studies and a review on the TMJ, characteristic MRI signals have been evaluated in the tops of the mandibular condyle in children. These structures were named “double contour-like structures” (DCLS) [1, 9–11]. Possible constituents of the structures in mandibular condyles have been speculated, and the significance of DCLS in the evaluation of TMJ growth in schoolchildren has been suggested [1, 9–11]. Moreover, in the present paper, it is demonstrated that the alteration period from red marrow to yellow marrow in the articular eminence of the temporal bone was earlier than that in the mandibular condyles on MRI.

## 2. MRI Findings of Mandibular Condyles in Adults and Schoolchildren

**Figure 1:**
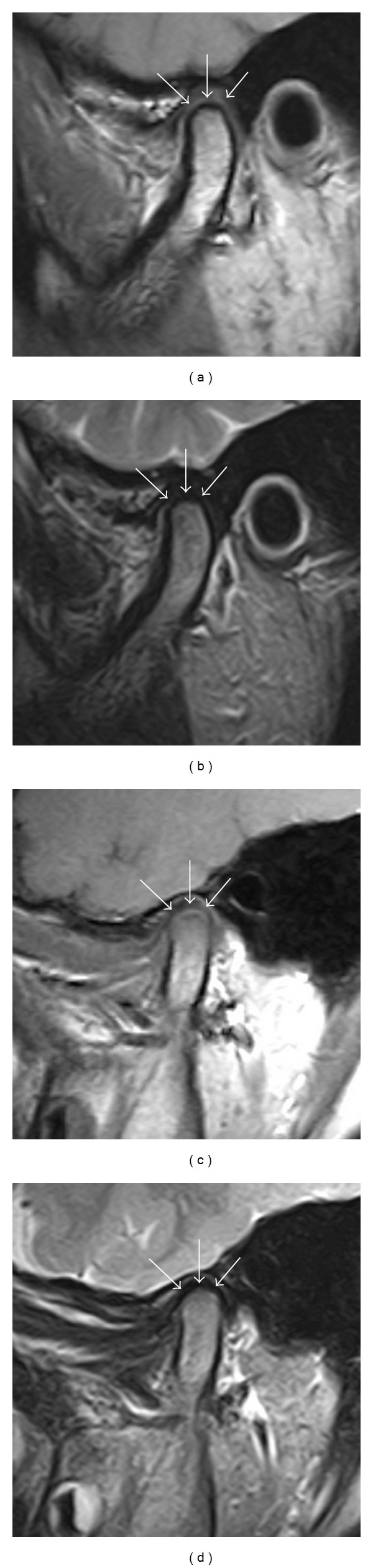
Differences in TMJ between normal adults (a, b) and schoolchildren (c, d) on MRI. (a) DCLS on the top of the mandibular condyles was undetectable (arrows) on fast spin-echo intermediate-weighted images of a healthy 34-year-old female. The voided signal line at the top of the right mandibular condyle was seen as a successive continuity. The imaging parameters used in the MR sequences included 1050 ms TR, 15 ms effective echo time, flip angle of 90°, a 15 × 15 cm field of view, a matrix of 160 × 288 pixels, 17 mm slab thickness, and 3 mm section thickness. (b) High-intensity stripes on the top of the mandibular condyles were undetectable (arrows) on fast spin-echo T2-weighted images of the same subject as Figure 1(a). The imaging parameters used in the MR sequences included 3500 ms TR, 108 ms effective echo time, flip angle of 90°, a 15 × 15 cm field of view, a matrix of 160 × 288 pixels, 17 mm slab thickness, and 3 mm section thickness. (c) DCLS on the top of the mandibular condyles (arrows) appeared as high-intensity stripes over a low-intensity area on fast spin-echo intermediate-weighted images of a healthy 8-year-old boy. The voided signal line at the top of the right mandibular condyle was not seen. The imaging parameters used in the MR sequences included 1050 ms TR, 15 ms effective echo time, flip angle of 90°, a 15 × 15 cm field of view, a matrix of 160 × 288 pixels, 17 mm slab thickness, and 3 mm section thickness. (d) High-intensity stripes (arrows) were found to coincide with DCLS on the top of the mandibular condyles; this was found using fast spin-echo T2-weighted images of the same subject as Figure 1(c). The imaging parameters used in the MR sequences included 3500 ms TR, 108 ms effective echo time, flip angle of 90°, a 15 × 15 cm field of view, a matrix of 160 × 288 pixels, 17 mm slab thickness, and 3 mm section thickness.

With the TMJ, differences in MRI findings are seen between adults and schoolchildren [1, 9–11]. In adults, in the sagittal plane at the center of the mandibular condyle, the TMJ disk is demonstrated as a biconcave structure with a relatively low signal intensity on fast spin-echo intermediate-weighted images (Figure 1(a)). The mandibular condyle is seen as a rounded eminence whose cortices have a voided signal intensity. This voided signal makes the outline of the mandibular condyles easily visible. The signal within the condyle is approximately the same as that of the fatty tissues. This is because bone marrow in the mandibular condyle converts from red bone marrow to yellow bone marrow after about 12–15 years of age [14, 15, 18–20]. As mentioned below, DCLS at the tops of mandibular condyles is undetectable. The top of the mandibular condyle is visualized as a rounded voided outline on T2-weighted images (Figure 1(b)). In the normal TMJ, synovial fluid in the cavity and status of retrodiscal tissue can be demonstrated on T2-weighted images. Synovial fluid in the TMJ cavity appears as linear and spot-like structures with high signal intensity (Figure 1(b)). However, the high-intensity stripes on the top of mandibular condyles were undetectable (Figure 1(b)).

In schoolchildren (aged 7–14 years) as well as in adults, the TMJ disk is demonstrated as a biconcave structure with a relatively low signal intensity (Figure 1(c)). However, the mandibular condyle is a rounded eminence whose cortices have an indistinct voided signal intensity. This voided signal means that the mandibular condyles are not easily visible. The signal within the condyle is intermediate in children aged 12–15 years (Figure 1(c)). In addition, structures on the tops of the mandibular condyles that appeared as high-intensity stripes over a crescent-like low-intensity area were identified on fast spin-echo intermediate-weighted images in schoolchildren (Figure 1(c)). These structures were also seen as high-intensity stripes on fast spin-echo T2-weighted images (Figure 1(d)). This characteristic structure was termed the “DCLS” by Tominaga and Morimoto, due to its strong similarity to MRI findings in patients with progressive bone remodeling, the so-called “double contour,” seen after intravertical ramus osteotomy (IVRO) and orthodontic treatments with an activator in adults [9, 17]. “Double contour” on MRI in adults, two voided signal bands sandwiching a high-signal region, was very clearly visualized [9, 17]. DCLS in schoolchild is also relatively similar to that, but two voided signal bands were relatively unclear. In addition, DCLS could not be detected with orthopantomography [9–11] which differs from DCLS. Of course, the DCLS also differs from the “double contour” that occurs after orthodontic treatments in adults after the use of an activator for the same reason [13]. The signal differences in the TMJ seen by MRI between adults and schoolchildren are similar to the MRI characteristics of the normal maturation of growing cartilage [2–8]. Some studies have demonstrated that high signal intensity changes are present within the distal femoral epiphyseal cartilage on intermediate-weighted MRI [2–4]. The top of the mandibular condyle is covered with articular cartilage, as is the knee joint [17, 21]. It is speculated that DCLS may correlate with findings in published reports of MRI of the epiphysis in the distal femoral epiphyseal cartilage in children [2–4]. High signal intensity changes may reflect more active ossification within the top of the mandibular condyle [2–4]. The increased signal intensity on MRI seen in the process of normal endochondral ossification may occur when cartilage cells undergo hypertrophy [4].

Moreover, the hyperintense stripe seen along the posterior surface of the distal femoral and proximal tibial metaphases in MRI, as described by Harcke et al., might also be very similar to DCLS [5]. DCLS most likely represents one part of the highly vascular loose fibrous tissue that encloses the periphery of the mandibular condyle, similar to the “hyperintense stripe” seen in the knee [5, 21, 22]. In particular, the characteristics of the hyperintense stripe are similar to those of the DCLS with respect to the disappearance with age in children aged 15 years, as mentioned below. In addition, DCLS appears to be different from the “double contour” after IVRO and is most likely not derived from bone formation [16, 23]. It is speculated that DCLS on MRI might be due to the synergistic effect of the two points described above [1, 9–11].

## 3. Alterations of DCLS on MRI according to Maturation of the TMJ in Adults

**Figure 2:**
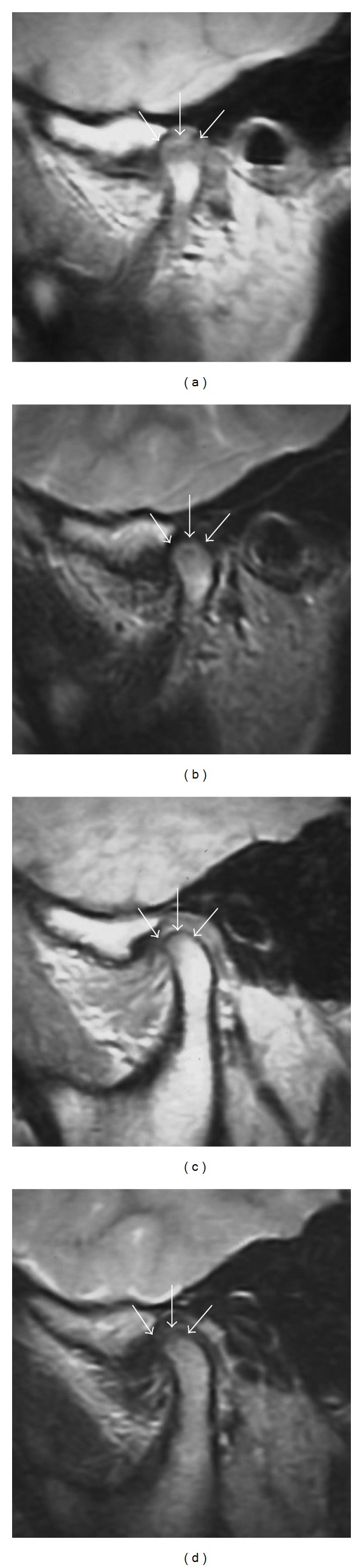
Alteration of mandibular condyles on MRI in growing schoolchildren over time. (a) DCLSs on the top of the mandibular condyles (arrows) appeared as high-intensity stripes over a low-intensity area on fast spin-echo intermediate-weighted images of a healthy 12-year-old boy at the first examination. The voided signal line at the top of the right mandibular condyle was not seen. The imaging parameters used in the MR sequences included 1050 ms TR, 15 ms effective echo time, flip angle of 90°, a 15 × 15 cm field of view, a matrix of 160 × 288 pixels, 17 mm slab thickness, and 3 mm section thickness. (b) High-intensity stripes (arrows) were found to coincide with DCLSs on the top of the mandibular condyles; this was found using fast spin-echo T2-weighted images of the same subject as Figure 1(a). The imaging parameters used in the MR sequences included 3500 ms TR, 108 ms effective echo time, flip angle of 90°, a 15 × 15 cm field of view, a matrix of 160 × 288 pixels, 17 mm slab thickness, and 3 mm section thickness. (c) DCLS at the top of the left mandibular condyle, apparent at the first examination, had disappeared by the second examination three years later in the same subjects as Figures 1(a) and 1(b). The imaging parameters used in the MR sequences included 1050 ms TR, 15 ms effective echo time, flip angle of 90°, a 15 × 15 cm field of view, a matrix of 160 × 288 pixels, 17 mm slab thickness, and 3 mm section thickness. (d) High-intensity stripes (arrows) were not seen at the top of the mandibular condyles at the second examination three years after the first one in the same subjects as Figures 1(a), 1(b), and 2(a). The imaging parameters used in the MR sequences included 3500 ms TR, 108 ms effective echo time, flip angle of 90°, a 15 × 15 cm field of view, a matrix of 160 × 288 pixels, 17 mm slab thickness, and 3 mm section thickness.

DCLS was detected in about 50% of TMJ mandibular condyles in consecutive studies in children [1, 9–11]. In children less than 12 years of age, the rate of DCLS seen on mandibular condyles with MRI increased, whereas the rate decreased in children over the age of 12 years [1, 9–11]. However, DCLS was undetectable in the TMJ of the youth and adults, as indicated above and seen in Figure 1. DCLS might disappear during the growth and development of the mandibular condyles. Therefore, in the same volunteers over time, it was evaluated whether or not DCLS in TMJ would be seen to alter on MRI [1, 9, 11]. It was found that DCLS disappeared as children grew [1, 9, 11]. DCLS at the top of the mandibular condyle was depicted as a high signal stripe over a low-signal streak on intermediate-weighted images at the first examination (Figure 2(a)). At the second examination three years later, DCLS had disappeared with advancing growth and development in the same volunteer on intermediate-weighted images (Figure 2(b)). On T2-weighted images, DCLS indicated as high-intensity stripes at the first examination (Figure 2(c)) had disappeared at the second examination (Figure 2(d)). As mentioned above, the disappearance of DCLS is similar to the hyperintense stripe seen along the posterior surface of the distal femoral and proximal tibial metaphases in MRI, as described by Laor et al. [24, 25]. The alternating appearance and disappearance of DCLS correlate with both the conversion of bone from red marrow to yellow marrow and eruption of the permanent maxillary second molars [9, 10]. At the same time, disappearance of DCLS is also related to the continuity of voided signal lines on the tops of the mandibular condyles on MRI and cortical bone-like radiopaque line on panoramic radiographs [11]. Radiopaque lines on panoramic radiographs and voided-signal continuity indicate a maturity of ossification in mandibular condyles [26]. After cartilage elimination treatment was applied to the tops of the mandibular condyles, the surface was still irregular in children aged 9–14 years but was smooth in adults [21, 22]. In other words, the conversion from a spongy bone-like state to a cortical bone-like state is apparent in the bone ossification style of the surface of the tops of mandibular condyles as children grow [21, 22]. Therefore, the possible constituents of the DCLS may be a complex of prolific cartilage and hypervascular fibrous tissues during the spongy bone-like state.

**Figure 3:**
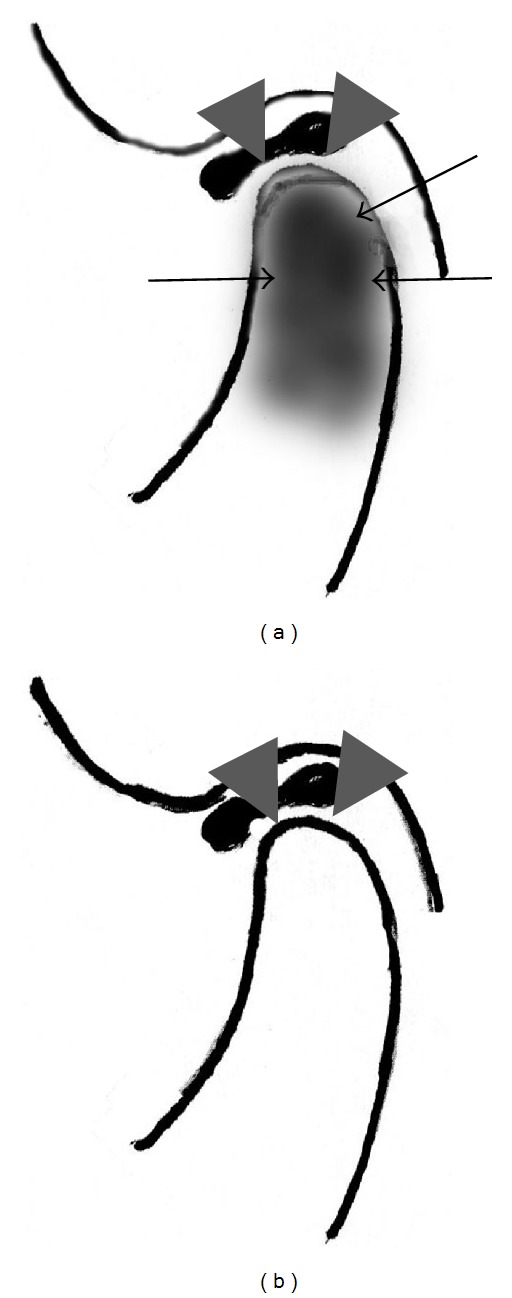
Scheme of the alternation of the top of mandibular condyle according to age. (a) In schoolchildren with DCLS, cancerous bone, hypervascular loose fibrous tissues, and proliferation of cartilage are laminating and conglomeritic (arrowheads). In addition, red marrow is inside the mandibular condyles (arrows). (b) In older subjects with disappearance of the DCLS, maturing bone (a cortical bone-like state), hypovascular loose fibrous tissues, and thinner cartilage are laminating (arrowheads).

In previous reports [1, 9–11] it has been speculated that constituents of the DCLS may be a laminating conglomeration of spongy bone, hypervascular loose fibrous tissues, and a proliferation of cartilage (Figure 3(a)). At the same time, red marrow is inside the mandibular condyles. Conversely, in subjects with disappearance of DCLS, maturing bone (the cortical bone-like state), hypovascular loose fibrous tissues, and a thinner cartilage are laminating (Figure 3(b)).

As for the clinical significance of DCLS, alterations in DCLS may be useful criteria for evaluating the staging of bone ossification in mandibular condyle, and maturation of the TMJ. The detection and disappearance of DCLS in mandibular condyles on MRI correlate with the eruption of the maxillary permanent second molars, conversion from red marrow to yellow marrow within the mandibular condyle, and voided signal continuity at the top of the mandibular condyle [1, 9–11]. Eruption of the maxillary permanent second molars means that the dental arch formed by permanent dentition and alveolar bone is almost completely developed [27, 28]. The normal conversion from hematopoietic to fatty marrow in the mandibular condyle represents maturation within the inside of the mandible [9, 27–29]. Radiopaque lines on panoramic radiographs and voided signal continuity indicate maturation of ossification of the outside of the mandibular condyle [30]. Therefore, the relationship between alterations of DCLS and these three aspects of the mandibular growth as mentioned above suggests that DCLS might directly correlate with growth of the mandibular condyle. The presence of DCLS on MRI may be a marker of the mandibular condyle growth spurt in the TMJ. For example, because the timing of the alternation of DCLS nearly coincides with the period of completion of the dental arch (indicated by the eruption of the permanent maxillary second molars), the present criterion may be useful in many cases [10, 11]. Presently, if alternation of DCLS seen by MRI was considered in addition to the present criteria regarding the beginning of orthodontic therapy, prevention of temporomandibular disorders (TMD) during and after orthodontic therapy might be possible.

## 4. Conversion from Red to Yellow Marrow between the Mandibular Condyles and the Articular Eminences and Glenoid Fossa

**Figure 4:**
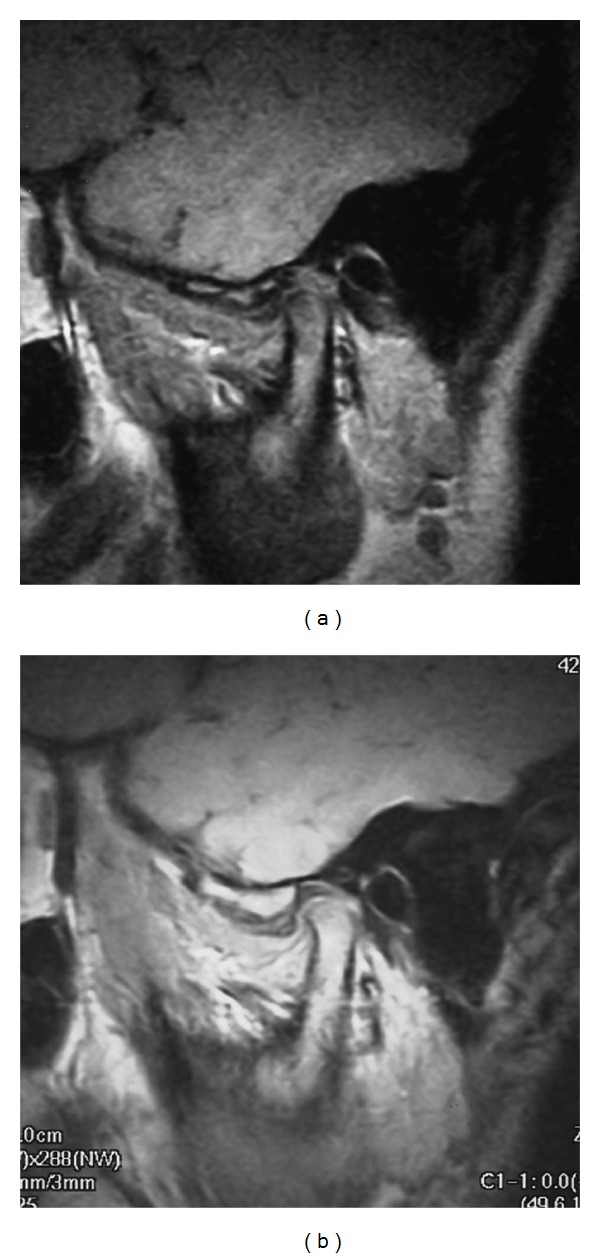
Differences in the MRI signal within the articular eminences and mandibular condyles. (a) Morphology of the articular eminences and glenoid fossa of TMJ on MRI is in conformity with the outline of the mandibular condyles on fast spin-echo intermediate-weighted images of a healthy 10-year-old boy at the first examination. MRI signal of the articular eminence was yellow marrow, but that of mandibular condyle was red. The imaging parameters used in the MR sequences included 1050 ms TR, 15 ms effective echo time, flip angle of 90°, a 15 × 15 cm field of view, a matrix of 160 × 288 pixels, 17 mm slab thickness, and 3 mm section thickness. (b) Morphology of the articular eminences and glenoid fossa of TMJ on MRI is in conformity with the outline of the mandibular condyles on the second examination three years after the first examination in the same subject as Figure 4(a). MRI signal of the articular eminence was of yellow marrow, and that of the mandibular condyle was also yellow marrow. The imaging parameters used in the MR sequences included 1050 ms TR, 15 ms effective echo time, flip angle of 90°, a 15 × 15 cm field of view, a matrix of 160 × 288 pixels, 17 mm slab thickness, and 3 mm section thickness.

Growth of the articular eminences and glenoid fossa in the TMJ of schoolchildren was also evaluated in addition to alterations of DCLS on the top of the mandibular condyles. Morphological alterations of articular eminences and glenoid fossa according to growth and development are connected to and correlate with those of mandibular condyles [18–20]. Certainly, the alteration of the morphology in articular eminences and glenoid fossa of TMJ on MRI correlates with the outline of mandibular condyles (Figure 4). However, timing of the conversion from red to yellow marrow inside of the articular eminence on MRI was seen to be earlier than that of the mandibular condyles (Figure 4) (unpublished data). MRI signals of the inside of the articular eminence in 38 (76 TMJ) schoolchildren (aged 9–15 years) were found to all consist of yellow marrow (Table 1). On the contrary, the conversion from red to yellow marrow in the mandibular condyles is %60–70% until 15 years of age and is 100% at 30 years of age, with a turning point of 11 years of age [12, 15]. Anatomy textbooks describe that growth and development in the articular eminence and glenoid fossa of the TMJ occur earlier than those in the mandibular condyle [18]. In fact, mandibular condyles are an assembly of undifferentiated mesenchymal cells during the period of growth of the glenoid fossa [19, 20], in agreement with the present findings. However, the present report did not include MRI of the TMJ in normal subjects less than six years of age. Therefore, the turning point of the conversion from red to yellow marrow in the articular eminence of TMJs could not be elucidated, and further study is needed.

## 5. Conclusion

In MR findings of mandibular condyles in schoolchildren, “DCLS” was visualized as the characteristic structure due to its strong similarity to MRI findings in patients with progressive bone remodeling, the so-called “double contour,” seen after IVRO. The timing of the appearances or disappearances of DCLS nearly coincides with the period of the completion of the dental arch (indicated by the eruption of the permanent maxillary second molars); the present criterion may be useful in many cases. The presence of DCLS on MRI may be a marker of the mandibular condyle growth spurt in the TMJ. Therefore, if the alternation of DCLS seen by MRI was considered in addition to the present criteria regarding the beginning of orthodontic therapy, prevention of temporomandibular disorders (TMD) during and after orthodontic therapy might be possible.

## Figures and Tables

**Table 1:** Conversion from red to yellow marrow in mandibular condyles and articular eminence on MRI.

Subjects' age (y)	Number of TMJs	Number of status of bone		Number of status of bone	
		marrow of mandibular condyles		marrow of articular eminence	
		Red	Yellow	Red	Yellow
9	6	5	1	0	6
10	22	18	4	0	22
11	4	3	1	0	4
12	22	16	6	0	22
13	8	2	6	0	8
14	8	2	6	0	8
15	6	1	5	0	6

	76	47	29	0	76

MRI: magnetic resonance imaging.

TMJ: temporomandibular joint.
